# 1-Allyl-3-amino-1*H*-pyrazole-4-carboxylic acid

**DOI:** 10.1107/S1600536808035538

**Published:** 2008-11-08

**Authors:** Gong-Chun Li, Li-Ye Wang, Ran Zhu, Feng-Ling Yang

**Affiliations:** a College of Chemistry and Chemical Engineering, Xuchang University, Xuchang, Henan Province 461000, People’s Republic of China; b State Key Laboratory of Elemento-Organic Chemistry, Nankai University, Tianjin 300071, People’s Republic of China

## Abstract

The title compound, C_7_H_9_N_3_O_2_, was prepared by alkaline hydrolysis of ethyl 1-allyl-3-amino-1*H*-pyrazole-4-carboxyl­ate. The crystal structure is stabilized by three types of inter­molecular hydrogen bond (N—H⋯O, N—H⋯N and O—H⋯N).

## Related literature

For details of the biological activities of pyrazole derivatives, see: Malhotra *et al.* (1997 ▶); Takao *et al.* (1994 ▶); Wang *et al.* (2005 ▶).
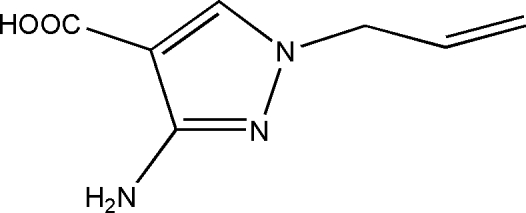


## Experimental

### 

#### Crystal data

C_7_H_9_N_3_O_2_
*M* *_r_* = 167.17
Monoclinic, 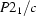

*a* = 8.966 (2) Å
*b* = 8.531 (2) Å
*c* = 10.266 (2) Å
β = 95.57 (3)°
*V* = 781.5 (3) Å^3^
*Z* = 4
Mo *K*α radiation
μ = 0.11 mm^−1^
*T* = 113 (2) K
0.20 × 0.18 × 0.14 mm

#### Data collection

Rigaku Saturn CCD area-detector diffractometer
Absorption correction: multi-scan (*CrystalClear*; Rigaku/MSC, 2005 ▶) *T*_min_ = 0.979, *T*_max_ = 0.985
5773 measured reflections
1852 independent reflections
1631 reflections with *I* > 2σ(*I*)
*R*_int_ = 0.025

#### Refinement

*R*[*F* ^2^ > 2σ(*F* ^2^)] = 0.034
*wR*(*F* ^2^) = 0.085
*S* = 1.06
1852 reflections
121 parameters
H atoms treated by a mixture of independent and constrained refinement
Δρ_max_ = 0.29 e Å^−3^
Δρ_min_ = −0.26 e Å^−3^

### 

Data collection: *CrystalClear* (Rigaku/MSC, 2005 ▶); cell refinement: *CrystalClear*; data reduction: *CrystalClear*; program(s) used to solve structure: *SHELXS97* (Sheldrick, 2008 ▶); program(s) used to refine structure: *SHELXL97* (Sheldrick, 2008 ▶); molecular graphics: *ORTEP-3* (Farrugia, 1997 ▶) and *DIAMOND* (Brandenburg, 1998 ▶); software used to prepare material for publication: *CrystalStructure* (Rigaku/MSC, 2005 ▶).

## Supplementary Material

Crystal structure: contains datablocks global, I. DOI: 10.1107/S1600536808035538/lx2076sup1.cif

Structure factors: contains datablocks I. DOI: 10.1107/S1600536808035538/lx2076Isup2.hkl

Additional supplementary materials:  crystallographic information; 3D view; checkCIF report

## Figures and Tables

**Table 1:** Hydrogen-bond geometry (Å, °)

*D*—H⋯*A*	*D*—H	H⋯*A*	*D*⋯*A*	*D*—H⋯*A*
N1—H1*A*⋯O1^i^	0.894 (16)	2.073 (16)	2.9652 (13)	175.7 (14)
N1—H1*B*⋯N2^ii^	0.905 (17)	2.457 (16)	3.2187 (14)	142.1 (13)
O2—H2*A*⋯N1^iii^	0.92 (2)	1.82 (2)	2.7232 (14)	166.8 (18)

Symmetry codes: (i) 
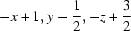
; (ii) 
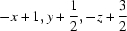
; (iii) 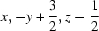.

**Fig. 1.**
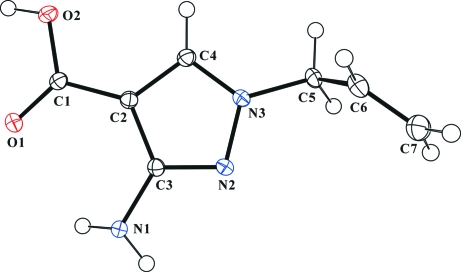
The molecular structure of the title compound, showing displacement ellipsoids drawn at the 50% probability level.

**Fig. 2.**
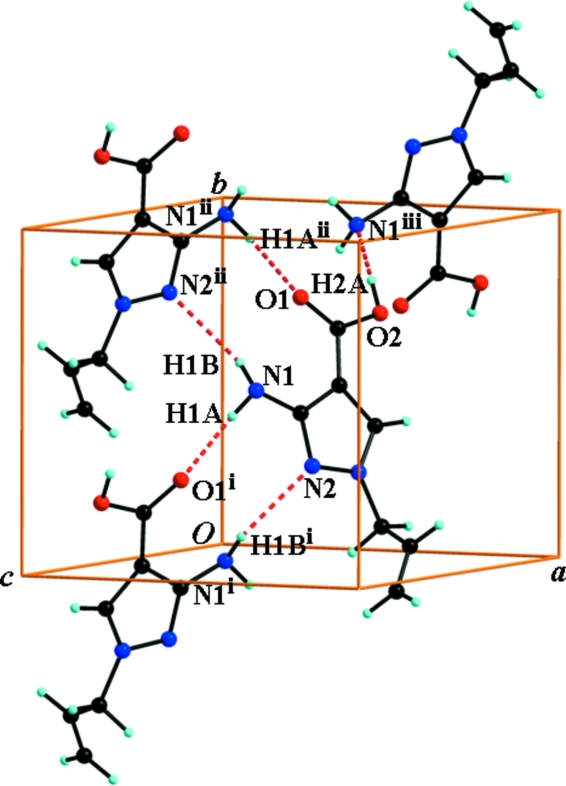
Hydrogenbonds interactions (dotted lines) in the title compound. [symmetry code; (i) -x+1, y-1/2, -z+3/2; (ii) -x+1, y+1/2, -z+3/2; (iii) x, -y+3/2, z-1/2.]

## supplementary crystallographic information

### Comment

Pyrazole ring derivatives are very important substances in biology and have many application in the field of pesticide and pharmaceutical agents (Malhotra *et al.*,  1997; Takao *et al.*,  1994). Some of these compounds such as pyrazosufuron have been sold as agrochemicals (Wang *et al.*, 2005).

Here we report the synthesis and crystal structure of the title compound, 1-allyl-3-amino-1H-pyrazole-4-carboxylic acid (Fig. 1). The crystal packing (Fig. 2) is stabilized by the intermolecular hydrogen bonds (Fig. 2 & Table 1).

### Experimental

The mixture of ethyl 1-allyl-3-amino-1H-pyrazole-4-carboxylate (1.95 g, 10 mmol) in THF-MeOH (50 ml, v/v = 1/1) with 2.5N NaOH(25 ml) was heated at 333 K for 4 h. The solvent was removed under reduced pressure and the residue was acidified with 6N  HCl at 273 K. A gray solid was precipitated, filtered, and washed with water. Single crystals suitable for X-ray diffraction were obtained by recrystallization of the title compound in ethanol.

### Refinement

H atoms of N1 and O2 were positioned in a difference Fourier maps and their parameters were freely refined. The other H atoms were placed in calculated positions, with C—H = 0.95  or 0.99 Å, and and O—H = 0.82 Å, and included in the final cycles of refinement using a riding model, with *U*_iso_(H) = 1.2Ueq(C).

### Crystal data

**Table:**

C_7_H_9_N_3_O_2_	*F*_000_ = 352
*M**_r_* = 167.17	*D*_x_ = 1.421 Mg m^−^^3^
Monoclinic, *P*2_1_/*c*	Mo *K*α radiation λ = 0.71073 Å
Hall symbol: -P 2ybc	Cell parameters from 2299 reflections
*a* = 8.966 (2) Å	θ = 2.4–27.9º
*b* = 8.531 (2) Å	µ = 0.11 mm^−^^1^
*c* = 10.266 (2) Å	*T* = 113 (2) K
β = 95.57 (3)º	Prism, colorless
*V* = 781.5 (3) Å^3^	0.20 × 0.18 × 0.14 mm
*Z* = 4	

### Data collection

**Table:**

Rigaku Saturn CCD area-detector diffractometer	1852 independent reflections
Radiation source: rotating anode	1631 reflections with *I* > 2σ(*I*)
Monochromator: confocal	*R*_int_ = 0.025
Detector resolution: 7.31 pixels mm^-1^	θ_max_ = 27.9º
*T* = 113(2) K	θ_min_ = 3.1º
ω and φ scans	*h* = −7→11
Absorption correction: Multi-scan(CrystalClear; Rigaku/MSC, 2005)	*k* = −11→11
*T*_min_ = 0.979, *T*_max_ = 0.985	*l* = −13→13
5773 measured reflections	

### Refinement

**Table:**

Refinement on *F*^2^	Secondary atom site location: difference Fourier map
Least-squares matrix: full	Hydrogen site location: inferred from neighbouring sites
*R*[*F*^2^ > 2σ(*F*^2^)] = 0.034	H atoms treated by a mixture of independent and constrained refinement
*wR*(*F*^2^) = 0.085	*w* = 1/[σ^2^(*F*_o_^2^) + (0.0353*P*)^2^ + 0.3376*P*] where *P* = (*F*_o_^2^ + 2*F*_c_^2^)/3
*S* = 1.06	(Δ/σ)_max_ < 0.001
1852 reflections	Δρ_max_ = 0.29 e Å^−^^3^
121 parameters	Δρ_min_ = −0.25 e Å^−^^3^
Primary atom site location: structure-invariant direct methods	Extinction correction: none

### Special details

**Table:**

Geometry. All esds (except the esd in the dihedral angle between two l.s. planes) are estimated using the full covariance matrix. The cell esds are taken into account individually in the estimation of esds in distances, angles and torsion angles; correlations between esds in cell parameters are only used when they are defined by crystal symmetry. An approximate (isotropic) treatment of cell esds is used for estimating esds involving l.s. planes.
Refinement. Refinement of F^2^ against ALL reflections. The weighted R-factor wR and goodness of fit S are based on F^2^, conventional R-factors R are based on F, with F set to zero for negative F^2^. The threshold expression of F^2^ > 2sigma(F^2^) is used only for calculating R-factors(gt) etc. and is not relevant to the choice of reflections for refinement. R-factors based on F^2^ are statistically about twice as large as those based on F, and R- factors based on ALL data will be even larger.

### Fractional atomic coordinates and isotropic or equivalent isotropic displacement parameters (Å^2^)

**Table:**

	*x*	*y*	*z*	*U*_iso_*/*U*_eq_	
C1	0.64833 (12)	0.69316 (12)	0.51583 (10)	0.0123 (2)	
C2	0.68026 (12)	0.53444 (13)	0.56126 (10)	0.0122 (2)	
C3	0.63332 (12)	0.46275 (12)	0.67507 (10)	0.0116 (2)	
C4	0.75001 (12)	0.41422 (13)	0.49875 (10)	0.0134 (2)	
H4	0.7935	0.4217	0.4182	0.016*	
C5	0.79757 (13)	0.12954 (13)	0.54567 (11)	0.0149 (2)	
H5A	0.8212	0.1241	0.4536	0.018*	
H5B	0.7169	0.0529	0.5570	0.018*	
C6	0.93408 (13)	0.08629 (14)	0.63419 (11)	0.0177 (2)	
H6	1.0204	0.1508	0.6340	0.021*	
C7	0.94070 (15)	−0.03654 (16)	0.71224 (12)	0.0238 (3)	
H7A	0.8560	−0.1030	0.7143	0.029*	
H7B	1.0302	−0.0587	0.7665	0.029*	
N1	0.55906 (11)	0.53395 (11)	0.77249 (9)	0.0132 (2)	
H1A	0.5169 (17)	0.4628 (18)	0.8213 (15)	0.024 (4)*	
H1B	0.4929 (18)	0.6087 (19)	0.7423 (15)	0.025 (4)*	
N2	0.67310 (10)	0.31234 (11)	0.68348 (9)	0.0128 (2)	
N3	0.74458 (10)	0.28673 (11)	0.57279 (9)	0.0128 (2)	
O1	0.57152 (9)	0.78485 (9)	0.57215 (8)	0.01648 (19)	
O2	0.70880 (9)	0.72908 (10)	0.40620 (8)	0.01749 (19)	
H2A	0.663 (2)	0.818 (2)	0.3719 (19)	0.049 (5)*	

### Atomic displacement parameters (Å^2^)

**Table:**

	*U*^11^	*U*^22^	*U*^33^	*U*^12^	*U*^13^	*U*^23^
C1	0.0129 (5)	0.0125 (5)	0.0113 (5)	−0.0016 (4)	0.0006 (4)	0.0004 (4)
C2	0.0130 (5)	0.0122 (5)	0.0114 (5)	0.0000 (4)	0.0007 (4)	0.0000 (4)
C3	0.0118 (5)	0.0113 (5)	0.0114 (5)	−0.0008 (4)	0.0001 (4)	−0.0012 (4)
C4	0.0141 (5)	0.0142 (5)	0.0119 (5)	0.0006 (4)	0.0016 (4)	0.0012 (4)
C5	0.0187 (5)	0.0114 (5)	0.0151 (5)	0.0026 (4)	0.0029 (4)	−0.0025 (4)
C6	0.0151 (5)	0.0168 (6)	0.0215 (5)	0.0026 (4)	0.0032 (4)	−0.0032 (4)
C7	0.0237 (6)	0.0262 (7)	0.0216 (6)	0.0075 (5)	0.0021 (5)	0.0031 (5)
N1	0.0170 (5)	0.0104 (4)	0.0127 (4)	0.0009 (4)	0.0043 (4)	0.0006 (3)
N2	0.0148 (4)	0.0124 (5)	0.0114 (4)	0.0007 (3)	0.0032 (3)	−0.0012 (3)
N3	0.0146 (4)	0.0124 (5)	0.0116 (4)	0.0015 (3)	0.0027 (3)	−0.0010 (3)
O1	0.0215 (4)	0.0125 (4)	0.0160 (4)	0.0026 (3)	0.0048 (3)	−0.0005 (3)
O2	0.0216 (4)	0.0160 (4)	0.0160 (4)	0.0043 (3)	0.0077 (3)	0.0058 (3)

### Geometric parameters (Å, °)

**Table:**

C1—O1	1.2238 (13)	C5—H5A	0.9900
C1—O2	1.3316 (13)	C5—H5B	0.9900
C1—C2	1.4516 (15)	C6—C7	1.3170 (17)
C2—C4	1.3902 (15)	C6—H6	0.9500
C2—C3	1.4182 (14)	C7—H7A	0.9500
C3—N2	1.3323 (14)	C7—H7B	0.9500
C3—N1	1.3936 (14)	N1—H1A	0.894 (16)
C4—N3	1.3305 (14)	N1—H1B	0.905 (17)
C4—H4	0.9500	N2—N3	1.3752 (13)
C5—N3	1.4585 (14)	O2—H2A	0.92 (2)
C5—C6	1.4980 (16)		
			
O1—C1—O2	123.11 (10)	C6—C5—H5B	109.2
O1—C1—C2	123.16 (10)	H5A—C5—H5B	107.9
O2—C1—C2	113.73 (9)	C7—C6—C5	123.43 (11)
C4—C2—C3	104.18 (9)	C7—C6—H6	118.3
C4—C2—C1	128.56 (10)	C5—C6—H6	118.3
C3—C2—C1	127.00 (10)	C6—C7—H7A	120.0
N2—C3—N1	121.12 (10)	C6—C7—H7B	120.0
N2—C3—C2	111.70 (9)	H7A—C7—H7B	120.0
N1—C3—C2	127.15 (10)	C3—N1—H1A	111.3 (10)
N3—C4—C2	107.24 (9)	C3—N1—H1B	113.8 (10)
N3—C4—H4	126.4	H1A—N1—H1B	111.8 (14)
C2—C4—H4	126.4	C3—N2—N3	104.05 (9)
N3—C5—C6	111.89 (9)	C4—N3—N2	112.83 (9)
N3—C5—H5A	109.2	C4—N3—C5	127.73 (9)
C6—C5—H5A	109.2	N2—N3—C5	119.37 (9)
N3—C5—H5B	109.2	C1—O2—H2A	108.1 (12)
			
O1—C1—C2—C4	171.64 (11)	N3—C5—C6—C7	121.64 (12)
O2—C1—C2—C4	−7.54 (16)	N1—C3—N2—N3	−178.90 (9)
O1—C1—C2—C3	−1.60 (17)	C2—C3—N2—N3	−0.81 (12)
O2—C1—C2—C3	179.22 (10)	C2—C4—N3—N2	0.52 (12)
C4—C2—C3—N2	1.12 (12)	C2—C4—N3—C5	177.29 (10)
C1—C2—C3—N2	175.67 (10)	C3—N2—N3—C4	0.18 (12)
C4—C2—C3—N1	179.07 (10)	C3—N2—N3—C5	−176.89 (9)
C1—C2—C3—N1	−6.38 (18)	C6—C5—N3—C4	109.64 (12)
C3—C2—C4—N3	−0.95 (12)	C6—C5—N3—N2	−73.77 (12)
C1—C2—C4—N3	−175.38 (10)		

### Hydrogen-bond geometry (Å, °)

**Table:**

*D*—H···*A*	*D*—H	H···*A*	*D*···*A*	*D*—H···*A*
N1—H1A···O1^i^	0.894 (16)	2.073 (16)	2.9652 (13)	175.7 (14)
N1—H1B···N2^ii^	0.905 (17)	2.457 (16)	3.2187 (14)	142.1 (13)
O2—H2A···N1^iii^	0.92 (2)	1.82 (2)	2.7232 (14)	166.8 (18)

Symmetry codes: (i) −*x*+1, *y*−1/2, −*z*+3/2; (ii) −*x*+1, *y*+1/2, −*z*+3/2; (iii) *x*, −*y*+3/2, *z*−1/2.
